# Influence of *TP53* Mutation on Survival of Diffuse Large B-Cell Lymphoma in the CAR T-Cell Era

**DOI:** 10.3390/cancers13225592

**Published:** 2021-11-09

**Authors:** Edit Porpaczy, Philipp Wohlfarth, Oliver Königsbrügge, Werner Rabitsch, Cathrin Skrabs, Philipp Staber, Nina Worel, Leonhard Müllauer, Ingrid Simonitsch-Klupp, Christoph Kornauth, Johannes Rohrbeck, Ulrich Jaeger, Ana-Iris Schiefer

**Affiliations:** 1 Department of Internal Medicine I, Division of Hematology and Hemostaseology, Medical University of Vienna, 1090 Vienna, Austria; edit.porpaczy@meduniwien.ac.at (E.P.); oliver.koenigsbruegge@meduniwien.ac.at (O.K.); cathrin.skrabs@meduniwien.ac.at (C.S.); philipp.staber@meduniwien.ac.at (P.S.); ulrich.jaeger@meduniwien.ac.at (U.J.); 2 Department of Internal Medicine I, Hematopoietic Stem Cell Transplantation Unit, Medical University of Vienna, 1090 Vienna, Austria; philipp.wohlfarth@meduniwien.ac.at (P.W.); werner.rabitsch@meduniwien.ac.at (W.R.); 3 Department of Blood Group Serology and Transfusion Medicine, Medical University of Vienna, 1090 Vienna, Austria; nina.worel@meduniwien.ac.at; 4 Department of Pathology, Medical University of Vienna, 1090 Vienna, Austria; leonhard.muellauer@meduniwien.ac.at (L.M.); ingrid.simonitsch-klupp@meduniwien.ac.at (I.S.-K.); christoph.kornauth@meduniwien.ac.at (C.K.); johannes.rohrbeck@meduniwien.ac.at (J.R.)

**Keywords:** DLBCL, anti-CD19 CAR T cells, *TP53* mutation, *TP53* polymorphism, overall survival

## Abstract

**Simple Summary:**

The genetic landscape of diffuse large B-cell lymphoma (DLBCL) is heterogenous. So far, detailed studies about *TP53* mutations in DLBCL treated with anti-CD19 chimeric antigen receptor T-cell (CAR T cells) therapy are still missing. Chemotherapy resistance is one of the challenges in *TP53* mutated tumors. New immunomodulatory agents, such as different inhibitors or CAR T cells, have shown durable responses in refractory/relapsed DLBCL in recent years. Although our CAR T cell treated cohort was small, we aimed to investigate the influence of *TP53* mutations on overall survival of patients treated with CAR T cells compared to DLBCL patients without CAR T-cell therapy. Identification of risk factors for treatment failure may aid in choosing the most promising treatment in every setting.

**Abstract:**

Refractory/relapsed diffuse large B-cell lymphoma (DLBCL) is associated with poor outcome. The clinical behavior and genetic landscape of DLBCL is heterogeneous and still not fully understood. *TP53* mutations in DLBCL have been identified as markers of poor prognosis and are often associated with therapeutic resistance. Chimeric antigen receptor T-cell therapy is an innovative therapeutic concept and represents a game-changing therapeutic option by supporting the patient’s own immune system to kill the tumor cells. We investigated the impact of *TP53* mutations on the overall survival of refractory/relapsed DLBCL patients treated with comparable numbers of therapy lines. The minimum number of therapy lines was 2 (median 4), including either anti-CD19 CAR T-cell therapy or conventional salvage therapy. A total of 170 patients with DLBCL and high-grade B-cell lymphoma with *MYC, BCL2,* and/or *BCL6* rearrangements (DHL/THL), diagnosed and treated in our hospital between 2000 and 2021, were included. Twenty-nine of them received CAR T-cell therapy. *TP53* mutations were found in 10/29 (35%) and 31/141 (22%) of patients in the CAR T-cell and conventional groups, respectively. Among the 141 patients not treated with CAR T cells, *TP53* mutation was an independent prognostic factor for overall survival (OS) (median 12 months with *TP53* vs. not reached without *TP53* mutation, *p* < 0.005), but in the CAR T cell treated group, this significance could not be shown (median OS 30 vs. 120 months, *p* = 0.263). The findings from this monocentric retrospective study indicate that *TP53* mutation status does not seem to affect outcomes in DLBCL patients treated with CAR T-cell therapy. Detailed evaluation in large cohorts is warranted.

## 1. Introduction

Diffuse large B-cell lymphoma (DLBCL) is an aggressive non-Hodgkin lymphoma (NHL) with heterogenous clinical behavior. Different genetic alterations influence clinical course and treatment response [1,2,3]. Besides genes involved in the B-cell receptor (BCR) signaling pathway, which is specific for lymphomas, other recurrent oncogenic mutations have been described in DLBCL [1,2,3,4]. Between five and ten percent of DLBCL have concurrent rearrangements of *MYC*, *BCL2,* and/or *BCL6* and are now recognized as a distinct entity in the 2016 WHO classification of lymphoid neoplasms as ‘high-grade B-cell lymphoma with *MYC*, *BCL2,* and/or *BCL6* rearrangements (DHL/THL)’.

*TP53* is a tumor suppressor gene and plays an important role in the cell cycle and proliferation. One of its main functions is to induce apoptosis in DNA damaged cells. Mutations in the *TP53* gene abrogate genetic stability and lead to uncontrolled proliferation of cancer cells [5]. *TP53* mutations are found in approximately 20–30% of DLBCL patients [2,6,7,8]. The role of the *TP53* mutation as a negative prognostic factor is well established in many malignancies, including DLBCL. Xu-Monette et al. have shown a predictive role for *TP53* in both the GCB and non-GCB DLBCL patients treated with rituximab, cyclophosphamide, doxorubicin, vincristine, and prednisolone (R-CHOP) [9].

Frequent occurrence of *TP53* alterations has been shown in DLBCL at relapse after therapy with R-CHOP, even if *TP53* mutations were absent at the initial diagnosis, suggesting a tendency towards mutations under immunochemotherapy [10].

Additionally, the presence of *TP53* mutations has been shown to be associated with drug resistance in several studies [11,12,13]. In DLBCL, *TP53* mutations are associated with dismal prognosis due to therapy refractoriness [7,9,10,14,15,16,17]. Several molecular mechanisms of drug resistance on the basis of *TP53* mutations have been described and were summarized by Hientz et al. [13].

The treatment of DLBCL is challenging. High-dose platin-containing agents in combination with other cytotoxic agents followed by autologous stem cell transplantation (ASCT) is still the established standard treatment to cure relapsed or refractory patients.

In recent years, chimeric antigen receptor T-cell (CAR T-cell) therapy has been shown to be a promising therapy in refractory/relapsed DLBCL [18,19]. CAR T-cell therapy has a completely different mechanism to eliminate tumor cells compared to cytotoxic agents. Patients’ autologous T cells are manufactured to gain the ability to target and kill lymphoma cells by gammaretroviral or lentiviral transduction of CARs against CD19, which is expressed on the surface of DLBCL cells. After infusion, CAR T cells recognize and destroy CD19-expressing cells. Compared with conventional salvage treatment, CAR T-cell therapy showed a clear benefit regarding survival in DLBCL [18]; however, more data from large trials are needed. Little is known about the impact of genetic alterations in lymphoma patients treated with CAR T cells.

The aim of the study was to investigate the impact of *TP53* mutations on overall survival in a cohort of patients with refractory/relapsed DLBCL and DHL/THL, comparing patients who received CAR T-cell therapy to control patients with conventional relapse therapies. Here we present the first preliminary data in a small DLBCL and DHL/THL cohort treated with anti-CD19 CAR T cells.

## 2. Materials and Methods

Patients: For this retrospective study, we included 170 patients with refractory/relapsed DLBCL and DHL/THL, treated either with anti-CD19 CAR T-cell therapy or as a control group treated with conventional therapy (N = 29 vs. 141). All patients received rituximab plus cyclophosphamide, doxorubicin, vincristine, and prednisone (R-CHOP) as first line therapy. In the control group, platin-containing regiments combined with rituximab were used in each case as second line salvage therapy. In the CAR T cell group, 22 patients received platin-based second line treatment; 7 patients received other second line treatments (bendamustine, gemcitabine, or venetoclax); and all 29 patients received combination therapy with antibodies. Third or later line therapies included antibodies (rituximab, obinotuzumab, polatuzumab), conventional cytotoxic agents (gemcitabine, bendamustine, pixantrone), and small molecule inhibitors (f.e., ibrutinib, idelalisib, revlimid, venetoclax, selinexor), alone or in combination with radiation. In the control group, none of the patients received CAR T cells at any time.

Inclusion criteria were: (1) refractory or relapsed DLBCL or DHL/THL with at least two previous therapy lines; (2) including rituximab-containing treatment; (3) diagnosed and treated at our institution since 2000; (4) 18 years of age or older; (5) known detailed medical history. Transplant- and human immunodeficiency virus (HIV)-associated lymphomas were excluded. Clinical data, including medical history, date of diagnosis, clinical stage [20] according to Ann-Arbor classification, cell of origin (COO) according to the Hans algorithm [21], double hit protein expression score (DHS), *TP53* mutations, *MYC* and *BCL2* translocations, type and duration (including number of cycles) of treatments, date and quality of response, date of relapse, date of death including cause of death, and observation time, were collected. All cases were reviewed by three independent pathologists, and diagnoses were stated according to the 2016 WHO classification [22]. Patients were followed until June 2021 or until death, whichever occurred first.

Immunohistochemistry (IHC): IHC was performed on formalin-fixed, paraffin-embedded sections on an automated platform (Leica Bond III Immunostainer, Leica Biosystems, Nussloch, Germany) using routine protocols. Antibody Clone DO-7, DAKO, Glostrup, Denmark was used for p53 staining, which was interpreted as positive if at least 30% of tumor cells were moderately to strongly positive in a sheetlike, diffuse pattern within the whole tumor tissue or at least within parts of the tumor. For BCL2 and MYC staining, the following antibodies were used: BCL2 (Clone 124, DAKO, DK-2600 Glostrup, Denmark); MYC (Clone E121, Epitomics, Burlingame, CA, USA). Cut-off values for BCL2 and MYC were 50% and 40%, respectively.

Interphase FISH analysis: FISH was performed on formalin-fixed, paraffin-embedded tissue according to manufacturer recommendations. The following probes were used: LSI MYC Dual Color, Break Apart Rearrangement Probe (Metasystems, Altlussheim, Germany); LSI BCL2 Dual Color, Break Apart Rearrangement Probe (Abbott, North Chicago, IL, USA); and LSI BCL6 Dual Color, Break Apart Translocation Probe (Kreatech, Leica Biosystems, Nussloch, Germany). In each case, a total of 200 interphase nuclei were counted. The cut-off level for positivity was determined at 10% of nuclei showing aberrant hybridization signals.

Sequencing of *TP53*: *TP53* mutation is associated with increased p53 protein expression, and *TP53* mutational status was assessed in all p53 IHC-positive cases in the control group. *TP53* sequencing was performed using an ABI BigDye^TM^ Terminator version 1.1 Cycle Sequencing Kit (Thermo Fisher Scientific, Waltham, MA, USA). Sequencing analyses covered exons 4 to 11 and flanking intron regions. Purified DNA fragments were run on an ABI 330 Genetic Analyzer. Sequences were analyzed using the SeqScape analysis software program versions 2.5 and 2.7 (Applied Biosystems, Thermo Fisher Scientific, Waltham, MA, USA).

NGS: Tumor tissues of patients receiving CAR T cells were analyzed by next-generation sequencing. DNA was extracted from paraffin-embedded tissue blocks with the QIAamp Tissue Kit^TM^ (Quiagen, Hilden, Germany). DNA quantification was performed with the Qubit Assay Kit (Thermo Fisher Scientific). The DNA library was prepared with multiplex polymerase chain reaction with the DNA Oncomine^TM^ Comprehensive Panel v3 (Ion Torrent, Thermo Fisher Scientific), covering 161 genes, including full gene *TP53* (a list of analyzed genes is provided in Supplementary Materials; Table S1). After barcode adaption and quantification of the DNA library (Ion TaqMan^TM^ Library Quantitation Kit, Thermo Fisher Scientific), template preparation was carried out with the Ion Chef^TM^ System, where template-positive Ion Sphere^TM^ Particles were generated and amplified and an Ion 530^TM^ Chip was loaded. Sequencing was performed with an Ion S5^TM^ Sequencer (Thermo Fisher Scientific). Sequencing data were analyzed using Variant Caller^TM^ and Ion Reporter^TM^ (both Thermo Fisher Scientific).

Statistical methods: Statistical analysis was performed using IBM SPSS Statistics 27.0.1.0 (IBM Corporation, Armonk, NY, USA) software. The distribution of categorical variables between groups was performed with the chi-squared test, and continuous variables were compared with the Mann–Whitney U test. Normality was assessed by a Q-Q plot. Kaplan–Meier analysis was performed to assess overall survival (OS) and a log-rank test for a comparison of survival between groups. The OS was calculated from the date of diagnosis to the date of all-cause death. We considered *p*-values (*p*) of < 0.05 (2-sided) statistically significant. Cases with unknown *TP53* status were excluded.

## 3. Results

### 3.1. Patients

All refractory/relapsed DLBCL and DHL/THL patients who received anti-CD19 CAR T cells (*N* = 29) at our institute were included in this study. The control group (*N* = 141) consisted of refractory/relapsed DLBCL and DHL/THL patients with at least two rituximab-containing therapy lines and known *TP53* status either within the framework of routine work-up or from our patient cohort already published [8]. Due to the known prognostic value of *TP53* mutation shown by data from the literature [10,16,17,23] and from our own study group [8], *TP53* mutational analyses have been frequently performed at our institute since 2010 as part of the diagnostic routine work-up for relapsed/refractory DLBCL and DHL/THL. Initially, 202 potentially eligible control patients were included. Sixty-one patients of the control group were excluded due to insufficient clinical information. We finally ended up with a definitive study cohort of 170 patients, 29 thereof treated with CAR T cells and 141 controls. Both cohorts comprised patients of comparable age (CAR T cell patients vs. control patients: median ages at diagnosis, 54.83 vs. 59.76 years, *p* = 0.123) and gender (44.8% females and 55.2% males vs. 41.8% females and 58.2% males, *p* = 0.838) distribution. Nine (31.0%) out of 29 vs. 47 (33.3%) out of 141 patients had primary refractory DLBCL in the CAR T cell group vs. controls. The control patients received 2–15 therapy lines (median 5); the CAR T cell group received 2–13 (median 3) prior to CAR T-cell therapy. Conventional treatment consisted of platin-containing therapy in the second line. In the CAR T cell group, nine (31.0%) patients had primary refractory disease, and 20 (68.96%) patients relapsed after achieving complete remission with R-CHOP. Forty-seven (33.3%) out of 141 patients had primary refractoriness, and 94 (66.6%) relapsed in the controls. The median time from initiation of the first salvage treatment to CAR T cell infusion was 10.7 months.

*TP53* mutations were found in 34.5% of the CAR T cell patients and in 22% of the controls, indicating enrichment of poor prognostic patients in the CAR T cell cohort. Furthermore, 7.9% of the CAR T cell patients and 56% of the control group died within the observation period.

Patient clinical and demographic characteristics are presented in Table 1.

### 3.2. Pathohistological Characteristics of the CAR T Cell Cohort

FISH analyses for *MYC*, *BLC2*, and *BCL6* rearrangement were available for 27 (out of 29) patient samples. Eight (27.6%) patients had rearrangement of the *MYC* gene; two (6.9%) patients had additional translocations of the *BCL2* gene and one (3.5%) of the *BCL6* gene locus. Two (6.9%) patients were triple hits with rearrangements of *MYC*, *BCL2*, and *BCL6*. According to the WHO classification [22], a total of five (17.2%) patients were, therefore, reclassified as aggressive B-cell lymphoma with *MYC, BCL2*, and/or *BCL6* rearrangements (DHL/THL).

Immunohistochemistry for the BCL2 and MYC proteins was performed in 26 out of 29 CAR T samples. Nineteen (65.5%) patients expressed both proteins according to a double hit expression score of 2 [23] (double expressor lymphoma (DEL)); 7 (24.1%) expressed only the BCL2 protein.

According to the Hans algorithm [21], 16 patients showed a non-GCB phenotype, and 11 were categorized as GCB type.

### 3.3. Pathohistological Characteristics of the Control Group

FISH was performed in 130 out of 141 tumor samples from the control group. Sixty-four (45.4%) tumors showed a *MYC* rearrangement; 29 (20.57%) samples thereof had an additional *BCL2* rearrangement (hence belonging to the DHL category); one lymphoma sample revealed translocations in *MYC*, *BCL2*, and *BCL6* (THL). Fifty-two (36.9%) tumors expressed both the MYC and BCL2 proteins (DEL). Fifty-six (39.7%) were positive for only one of either MYC or BCL2. In detail, 66 (46.8%) patients expressed MYC and 100 (70.9%) patients the BCL2 protein. Forty-seven (33.3%) samples belonged to the non-GCB group; 82 (58.2%) were categorized as GCB. According to the Hans algorithm [21], 47 patients showed a non-GCB phenotype, and 82 were categorized as GCB type.

### 3.4. TP53 Mutations

From the CAR T cell cohort, tumor samples from 22 patients were sequenced by NGS. Analyses were done from the latest biopsy sample taken immediately before the start of treatment. From the remaining seven patients, material was not sufficient for molecular analyses.

Within the control group, screening for *TP53* mutations was performed by immunohistochemistry, and patients with >30% strongly positive tumor cells were further sequenced: 54 by Sanger sequencing covering exons 4–11, seven patient samples by NGS.

In summary, *TP53* mutations were detected in 41 patients and 44 patient samples (three patients from the control group had two concordant *TP53* mutations in different exons (Table 2)).

*TP53* mutations were found in 10 (34.5%) of the CAR T cell tumors and in 31 (22%) patients from the control group, respectively.

Within the DHL/THL patient group, *TP53* mutations were detectable in three (60%) out of five DHL/THLs in the CAR T cell cohort (one sample did not reveal *TP53* mutation by NGS, and in one sample, sequencing could not be performed). From the 16 DHL/THL (out of 30) samples within the control group, *TP53* mutations were detected in nine patients (56.3%).

The great majority of the mutations detected were missense mutations (*N* = 39, 95.1%), followed by four (9.8%) nonsense mutations resulting in truncated proteins; one (2.4%) sample from the CAR T cell cohort had an in-frame deletion of 3 bases in exon 4. Except for one variant, all mutations were found in the DNA-binding domain, the majority in exon 7 (*N* = 15, 36.6%). The most common mutation was R248Q (*N* = 5, 12.2%).

### 3.5. Influence of TP53 Mutations on Overall Survival

The mean observation period (date of diagnosis to last follow-up) was 49.49 months (range: 1 month to 321 months; standard deviation: 53.167 months).

**Figure 1:**
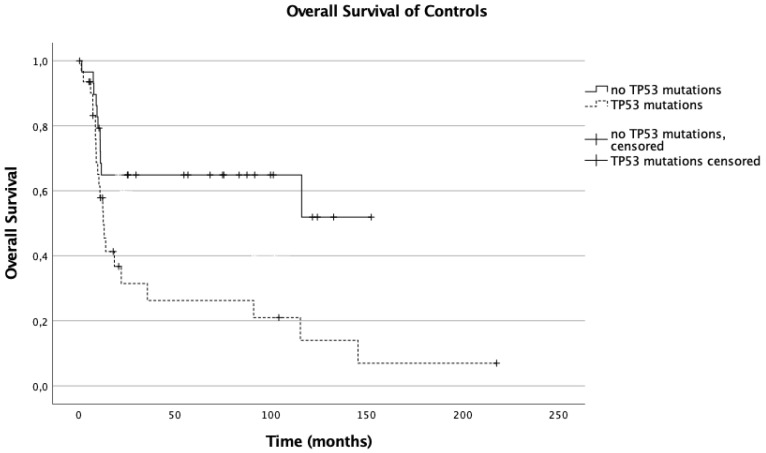
Overall survival of controls.

**Figure 2:**
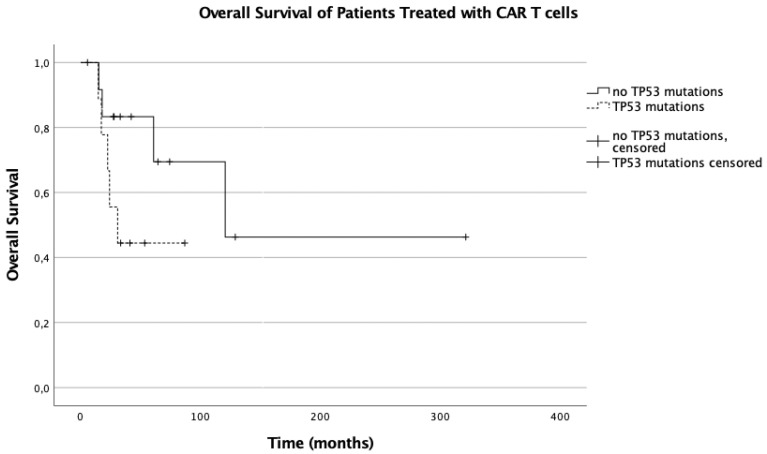
Overall survival of patients treated with CAR T cells.

Among the controls, the presence of *TP53* mutations was associated with a significantly (*p* = 0.005) worse overall survival (median OS with *TP53* mutations: 12.55 months, 95% confidence interval (95% CI): 9.167–15.933 vs. not reached with *TP53* wild type). This influence of *TP53* mutations could not be shown in the CAR T cell treated patients (median survival: 30.88 vs. 120.640 months, *p* = 0.263) (Figure 1 and Figure 2).

## 4. Discussion

DLBCL is a heterogenous disease with complex biological behavior. Relapsed or refractory DLBCL is associated with poor survival, and despite the rapid development of novel treatment strategies, management of those patients is still challenging for physicians. The negative prognostic impact of *TP53* mutation and its association with drug resistance is well known in many malignancies, including DLBCL [10,15]; however, the prognostic value of *TP53* mutations in refractory DLBCL patients receiving CAR T cells is still undefined. In this study, comprising only a small cohort of patients receiving CAR T cells, *TP53* lost its significance as a negative prognostic marker, indicating that CAR T cells might equalize—at least to some extent—the adverse effect of *TP53* mutations.

*TP53* has been one of the best described tumor suppressor genes since its recognition in 1989. A nonsynonymous *TP53* mutation is the most common reason for inactivation of p53. Point mutations comprise over 90% of *TP53* alterations in hematological malignancies; almost 80% are missense mutations. In our study, missense mutations even accounted for 95% of detected *TP53* alterations.

*TP53* displays its function via transcription-dependent and -independent activation. For both mechanisms, the DNA-binding domain plays an important role. Thus, most *TP53* deleterious mutations occur in the DNA-binding domain, which covers exons 4–8. Except one variant, all mutations in our study were found in the DNA-binding domain, the majority thereof in exon 7 (*N* = 15). The most common mutation was R248Q (*N* = 5), followed by R273H, the latter found in three tumor samples. The codons 248 and 273 bind to major and minor grooves of DNA and belong to the *TP53* mutation hot spots found in most human malignancies. The effect on p53 transcription function can be determined from the International Agency for Research on Cancer (IARC) *TP53* database [24]. Accordingly, 34 of the missense mutations had no transactivation function; two were partially functional, and three were functional. This is in line with data from the literature, where the vast majority of *TP53* mutations are classified as loss of function mutations in DLBCL [9].

Besides the cancer preventing role of *TP53*, its involvement in chemotherapy resistance has been described in many *TP53*-mutated tumors, causing dismal prognosis [25,26,27,28,29,30,31,32,33,34,35,36,37]. Even polymorphisms of *TP53* have been shown to be involved in drug resistance [29]. In DLBCL, little is known about the biological effect of *TP53* polymorphism. Of note, many of our patients had the *TP53* polymorphism R72P either as a sole aberration (N = 21) or concurrent with an actual pathogenic *TP53* mutation (N = 22, data not shown). Due to its yet unknown significance, this polymorphism was assigned as negative for *TP53* mutation in our study. Chemotherapy resistance is a considerable problem in refractory lymphomas. Before the availability of CAR T cell therapy and other biological treatments such as BTK- and other inhibitors, relapsed and refractory DLBCL patients had very limited therapy options, e.g., high-dose platin-containing regiments followed by autologous stem cell transplantation and, in rare cases, allogeneic stem cell transplantation where available for fit patients. Patients not eligible for high-dose chemotherapy were usually treated only in a palliative setting, with the goal to decelerate tumor progression and to raise life expectancy. For patients that demonstrate refractory disease to second line chemoimmunotherapy, outcomes have historically been dismal [38,39]. Even allogeneic transplantation performs very poorly if patients are not responding at the time of transplant. CAR T-cell therapy, on the other hand, is effective even in chemorefractory disease, which is a significant advantage. However, whether *TP53* status affects CAR T cell outcomes in DLBCL is unknown. The implementation of CAR T cells has revolutionized the therapeutic landscape in DLBCL. Compared with conventional chemotherapeutics, CAR T cells operate on a completely different basis. Besides the direct cytotoxic activity, CAR T cells have been shown to modulate the tumor microenvironment [40,41]. It is conceivable that treatment with CAR T cells might overcome the adverse effect of *TP53* mutations.

Many trials of CAR T-cell therapy have shown strong and durable responses in relapsed/refractory DLBCL disease [19,20,42,43]. Three FDA approved CAR T cell products (axicabtagene ciloleucel, tisagenlecleucel, and lisocabtagene maraleucel) are currently available for r/r DLBCL: axi-cell with an overall response (OR) rate of 83% (58% complete remission (CR) rate) [20] and tisa-cell with an OR of 52% and a CR rate of 40% [43]. Currently, many studies worldwide are investigating CAR T-cell treatment in DLBCL in different settings, including comparison of both autologous stem cell and CAR T-cell therapies in relapsed/refractory lymphomas.

The prognostic value of *TP53* mutations in refractory DLBCL patients receiving CAR T cells is still undefined. There are also only limited data on the efficacy of CAR T-cell therapy in patients with *TP53* mutations in another B-cell neoplasm, namely, B-ALL, where this novel treatment was first implemented. In a phase 1/2 CAR T-cell therapy study comprising 115 patients with refractory/relapsed B-ALL, *TP53* mutation was an independent indicator of poor prognosis. OS and leukemia-free survival (LFS) at 6 months were significantly lower in *TP53* mutated patients (OS: 51.9% vs. 89%; LFS: 42.4% vs. 82.6%) [44]. However, in the same study, no differences in CR rates at day 30 after CAR T-cell therapy were detected, indicating susceptibility of lymphoma/leukemia cells for CAR T cells. Similar results were obtained by Pan et al. [45]. CAR T-cell therapy was indeed able to overcome the adverse impact of *TP53* mutation to induce remission [46,47]; however, DFS and relapse rate were negatively influenced by *TP53* mutation [45]. *TP53* mutations were detected in 25.9% (14/56) of r/r B-ALLs treated with CD19 CAR T cells followed by consolidation with allogeneic hematopoietic stem cell transplantation or CD22 CAR T-cell infusion. The frequency of *TP53* mutation at relapse after CD19 CAR T-cell therapy was 66.7% (8/12). The anti-CD19 chimeric antigen receptor T-cell therapy KTE-X19 has shown efficacy in in patients with relapsed or refractory mantle-cell lymphoma, even in subgroups with high-risk features. *TP53* mutated patients (*N* = 6) had objective response rates comparable to *TP53* unmutated patients (*N* = 30) [48].

*TP53* mutations were more frequent in our CAR T cell cohort (34.5% vs. 22% in the control group), which was probably due to a bias in selection of heavily pretreated r/r DLBCL patients. On the other hand, our control group also consisted of r/r DLBCL patients with at least two rituximab-containing therapy lines. However, *TP53* mutated patients in the CAR T cell cohort trended towards worse outcomes compared with CAR T cell treated, *TP53* unmutated ones. The loss of significance regarding OS in the *TP53+* CAR T cell group (*p* = 0.263 vs. *p* = 0.005) was surprising. Control patients with *TP53* mutations had a median OS of 12.55 months, while patients treated with anti-CD19 CAR T cells had an OS 2.5 times longer (30.88 months).

Although our study cohort was too small to draw definitive conclusions, our findings may contribute to the understanding of the biology of DLBCL and may aid in choosing the most promising treatment in every setting.

We conclude that screening for *TP53* mutations in lymphomas might be important to identify potential non-responders to chemotherapeutic agents. In those cases, an early switch to therapy strategies using treatments with alternative mechanisms, such as CAR T cells, might help to improve prognosis.

We are conscious of the limitations of this study. As a retrospective analysis, the present study is prone to selection and reporting biases. Prospective randomized observational studies should be performed; however, ethical aspects must be considered. Further, the statistical power was limited due to the low sample size, especially of the CAR T cell cohort. The observation time was also still limited due to the novelty of CAR T-cell therapy and its limited availability in smaller centers. In the control group, only patients with positive p53 immunohistochemistry were further sequenced. Despite the reasonable and published correlation of p53 protein overexpression and *TP53* mutation [17], it is possible that some *TP53* mutated cases were missed in the control group. However, the number is expected to be low, also regarding the incidence of 22%, which is in line with published data.

## 5. Conclusions

Despite all of the limitations of this study, this is one of the first studies showing the impact of *TP53* mutation status on outcomes of patients treated with CAR T-cell therapy as opposed to conventional chemoimmunotherapy. Albeit in limited numbers (and only hypothesis generating), *TP53* mutation status does not seem to affect outcomes in DLBCL patients treated with CAR T-cell therapy. Our data suggest that *TP53* mutation analysis should be included when prognostic factors are evaluated before CAR T-cell treatment, because data regarding molecular prognostic markers of CAR T cell treated patients are very rare to date. The prognostic value of known genetic markers might be modified by novel therapies targeting other cellular mechanisms than cytotoxic agents. The rapid development of novel treatment strategies urges large multicenter studies comparing biomarkers for novel immunotherapies.

## Figures and Tables

**Table 1:** Patient characteristics.

Characteristics	Whole Cohort		CAR T cells		Controls		
	No	%	No	%	No	%	*p*-Value
	170	100	29	17.1	141	82.9	
Gender							
Female	72	42.4	13	44.8	59	41.8	0.838
Male	98	57.6	16	55.2	82	58.2	0.838
Median age at diagnosis	56.99	(19–95)	54.83	(31–79)	59.76	(19–95)	0.123
Stage = III–IV	107	62.9	10	34.5	97	68.8	<0.001
Extranodal	109	64.1	10	34.5	99	70.2	<0.001
Transformed	21	12.4	5	17.2	16	11.3	0.553
COO							
Non-GCB	63	36.8	16	55.2	47	33.3	0.035
GCB	93	55	11	37.9	82	58.2	0.064
NA	14	8.2	2	6.9	12	8.5	0.999
DEL	71	41.8	19	65.5	52	36.9	0.003
*TP53+*	41	24.1	10	34.5	31	22.0	0.804
*MYC*+	72	42.4	8	27.6	64	45.4	0.055
*BCL2+*	41	24.1	4	13.8	37	26.2	0.121
DHL/THL	35	20.6	5	17.2	30	21.3	0.628
Primary refractory DLBCL	56	32.9	9	31.0	47	33.3	0.833
Relapsed DLBCL	114	67.1	20	69.0	94	66.7	0.833
Death	90	52.9	11	37.9	79	56.0	0.102

COO: cell of origin; non-GCB: non-germinal center B-cell-like; GCB: germinal center B-cell-like; NA: not applicable; DEL: double expressor lymphoma (BCL2 and MYC protein expression); *TP53+*: presence of *TP53* mutation; *MYC+*: presence of *MYC* translocation; *BCL2+*: presence of *BCL2* translocation; DHL/THL: double hit lymphoma/triple hit lymphoma. The *p*-values for comparison of distributions were calculated with the Mann –Whitney U test for continuous variables and chi-squared test for categorical variables.

**Table 2:** *TP53* Mutations.

Diagnosis	Cohort	Sequencing	Mutation	Exon	Effect	TA Class
DLBCL	Control	Sanger	G266V	Exon8	Missense	Nonfunctional
DLBCL	Control	Sanger	Y205C	Exon6	Missense	Nonfunctional
DHL	Control	Sanger	M237I	Exon7	Missense	Nonfunctional
DLBCL	Control	Sanger	G245A	Exon7	Missense	Nonfunctional
DHL	Control	Sanger	F134L	Exon5	Missense	Nonfunctional
DLBCL	Control	Sanger	V143E	Exon5	Missense	Nonfunctional
DLBCL	Control	Sanger	R248Q	Exon7	Missense	Nonfunctional
			R267W	Exon8	Missense	Nonfunctional
DHL	Control	Sanger	R248Q	Exon7	Missense	Nonfunctional
DLBCL	Control	Sanger	R280S	Exon8	Missense	Nonfunctional
DLBCL	Control	Sanger	L194P	Exon6	Missense	Nonfunctional
			M246V	Exon7	Missense	Nonfunctional
DHL	Control	Sanger	R248Q	Exon7	Missense	Nonfunctional
DLBCL	Control	Sanger	R248Q	Exon7	Missense	Nonfunctional
DLBCL	Control	Sanger	G245S	Exon7	Missense	Nonfunctional
DLBCL	Control	Sanger	Y234S	Exon7	Missense	Nonfunctional
DLBCL	Control	Sanger	R273H	Exon8	Missense	Nonfunctional
DLBCL	Control	Sanger	Q331X	Exon9	Nonsense	Na
DLBCL	Control	Sanger	Y220C	Exon6	Missense	Nonfunctional
DLBCL	Control	Sanger	Y236H	Exon7	Missense	Nonfunctional
DLBCL	Control	Sanger	G245S	Exon7	Missense	Nonfunctional
DHL	Control	Sanger	W53L	Exon4	Missense	Functional
DHL	Control	Sanger	E286G	Exon8	Missense	Nonfunctional
DLBCL	Control	Sanger	R175H	Exon5	Missense	Nonfunctional
			G262V	Exon8	Missense	Nonfunctional
DLBCL	Control	Sanger	N239D	Exon7	Missense	Nonfunctional
DLBCL	Control	Sanger	H179Y	Exon5	Missense	Partially Functional
DHL	Control	NGS	R273H	Exon8	Missense	Nonfunctional
THL	Control	NGS	R175H	Exon5	Missense	Nonfunctional
DLBCL	Control	NGS	H214R	Exon6	Missense	Nonfunctional
DLBCL	Control	Sanger	G187S	Exon5	Missense	Functional
DLBCL	Control	Sanger	S315F	Exon9	Missense	Functional
DHL	Control	Sanger	V272E	Exon8	Missense	Nonfunctional
DLBCL	Control	Sanger	R282W	Exon8	Missense	Nonfunctional
THL	CAR T	NGS	V157G	Exon5	Missense	Nonfunctional
DLBCL	CAR T	NGS	C242S	Exon7	Missense	Nonfunctional
THL	CAR T	NGS	C137W	Exon5	Missense	Partially Functional
DLBCL	CAR T	NGS	R248Q	Exon7	Missense	Nonfunctional
DLBCL	CAR T	NGS	C176*	Exon5	Nonsense	na
DHL	CAR T	NGS	Y234C	Exon7	Missense	Nonfunctional
DLBCL	CAR T	NGS	Q167*	Exon5	Nonsense	na
DLBCL	CAR T	NGS	R273H	Exon8	Missense	Nonfunctional
DLBCL	CAR T	NGS	F113del	Exon4	Na	Na
DLBCL	CAR T	NGS	Q331*	Exon9	Nonsense	Na

DLBCL: diffuse large B-cell lymphoma; DHL: double hit lymphoma (*MYC* and *BCL2* or *BCL6* translocation); THL: triple hit lymphoma (*MYC + BCL2 + BCL6* translocation); NGS: next generation sequencing, TA: transactivational activity, NA: not applicable.

## Data Availability

Data are contained within the article.

## Supplementary Materials

The following are available online at https://www.mdpi.com/article/10.3390/cancers13225592/s1, Table S1: List of gene targets for detection of relevant SNVs, CNV, gene fusions and indels from 161 genes.
