# Supplementary material for: Influence of TP53 Mutation on Survival of Diffuse Large B-Cell Lymphoma in the CAR T-Cell Era
Source: Cancers (Basel). 2021 Nov 9;13(22):5592. doi: 10.3390/cancers13225592 (PMC8616128; doi:10.3390/cancers13225592)
Supplement: Supplementary file 1 [file cancers-13-05592-s001.zip › Supplementary Table S1.pdf]

Table S1. List of gene targets for detection of relevant SNVs, CNV, gene fusions and indels from 161.

**Hotspot genes**<br>***n*=87**	**Full length genes**<br>***n*=48**	**Copy number genes**<br>***n*=47:**	**Gene fusions**<br>***n*=51:**
AKT1 AKT2 AKT3 ALK<br>AR ARAF AXL BRAF BTK<br>CBL CCND1 CDK4 CDK6<br>CHEK2 CSF1R CTNNB1<br>DDR2 EGFR ERBB2<br>ERBB3 ERBB4 ERCC2<br>ESR1 EZH2 FGFR1 FGFR2<br>FGFR3 FGFR4 FLT3<br>FOXL2 GATA2 GNA11<br>GNAQ GNAS H3F3A<br>HIST1H3B HNF1A HRAS<br>IDH1 IDH2 JAK1 JAK2<br>JAK3 KDR KIT KNSTRN<br>KRAS MAGOH MAP2K1<br>MAP2K2 MAP2K4 MAPK1<br>MAX MDM4 MED12 MET<br>MTOR MYC MYCN<br>MYD88 NFE2L2 NRAS<br>NTRK1 NTRK2 NTRK3<br>PDGFRA PDGFRB PIK3CA<br>PIK3CB PPP2R1A PTPN11<br>RAC1 RAF1 RET RHEB<br>RHOA ROS1 SF3B1<br>SMAD4 SMO SPOP SRC<br>STAT3 TERT TOP1 U2AF1<br>XPO1	ARID1A ATM ATR ATRX<br>BAP1 BRCA1 BRCA2<br>CDK12 CDKN1B CDKN2A<br>CDKN2B CHEK1 CREBBP<br>FANCA FANCD2 FANCI<br>FBXW7 MLH1 MRE11A<br>MSH2 MSH6 NBN NF1 NF2<br>NOTCH1 NOTCH2<br>NOTCH3 PALB2 PIK3R1<br>PMS2 POLE PTCH1 PTEN<br>RAD50 RAD51 RAD51B<br>RAD51C RAD51D RB1<br>RNF43 SETD2 SLX4<br>SMARCA4 SMARCB1<br>STK11 TP53 TSC1 TSC2	AKT1 AKT2 AKT3 ALK AR<br>AXL BRAF CCND1 CCND2<br>CCND3 CCNE1 CDK2<br>CDK4 CDK6 CDKN2A<br>CDKN2B EGFR ERBB2<br>ESR1 FGF19 FGF3 FGFR1<br>FGFR2 FGFR3 FGFR4 FLT3<br>IGF1R KIT KRAS MDM2<br>MDM4 MET MYC MYCL<br>MYCN NTRK1 NTRK2<br>NTRK3 PDGFRA PDGFRB<br>PIK3CA PIK3CB PPARG<br>RICTOR TERT TSC1 TSC2	AKT2 ALK AR AXL BRAF<br>BRCA1 BRCA2 CDKN2A<br>EGFR ERBB2 ERBB4 ERG<br>ESR1 ETV1 ETV4 ETV5<br>FGFR1 FGFR2 FGFR3 FGR<br>FLT3 JAK2 KRAS MDM4<br>MET MYB MYBL1 NF1<br>NOTCH1 NOTCH4 NRG1<br>NTRK1 NTRK2 NTRK3<br>NUTM1 PDGFRA PDGFRB<br>PIK3CA PPARG PRKACA<br>PRKACB PTEN RAD51B<br>RAF1 RB1 RELA RET<br>ROS1 RSPO2 RSPO3 TERT

SNV: single nucleotide variant, CNV: copy number variation, indel: insertion- deletion mutation
